# Sleep Quality in Pamukkale University Students and its relationship with smartphone addiction

**DOI:** 10.12669/pjms.37.1.3130

**Published:** 2021

**Authors:** Betul Ozcan, Nurhan Meydan Acimis

**Affiliations:** 1 Betul Ozcan, MD Public Health Professional, Provincial Health Directorate, Agri Turkey; 2 Nurhan Meydan Acimis, Department of Public Health, Pamukkale University School of Medicine, Denizli Turkey

**Keywords:** Cell phone, Sleep, Students

## Abstract

**Objective:**

Research shows that poor sleep quality and smartphone addiction are common problems among university students. This study was planned to evaluate the quality of sleep in students at Pamukkale University and to investigate its relationship with smartphone addiction.

**Methods:**

This cross-sectional study was carried out at the university campus in 2017-2018. Its dependent variable was low sleep quality. Independent variables were smartphone addiction, features related to smartphone addiction, socio-demographic features and other questioned features. The total number of students attending normal education in seven faculties and two colleges for four years was 20862. The minimum sample size of the study was calculated as 1088. Smartphone Addiction Scale-Short Version (SAS-SV) and Pittsburgh Sleep Quality Index (PSQI) were used. The data were analyzed with the SPSS program.

**Results:**

The mean age was of the participants 21.39 ± 2.21. The sleep quality of students with a PSQI total score of more than five was defined as ‘poor’. The frequency of poor sleep quality was 52.4%. The frequency of smartphone addiction was 34.6% according to the SAS-SV scale. It was found that the frequency of poor sleep quality was significantly higher in students with smartphone addiction compared to others.

**Conclusion:**

Smartphone addiction was found as one of the risk factors for poor sleep quality.

## INTRODUCTION

Lifestyle changes and environmental factors are increasingly affecting sleep quality.^1^ University students may have different sleep problems from their peers due to their lifestyles and academic responsibilities.^2^,^3^ Research shows that having poor sleep quality and smartphone addiction is a common problems among university students.^4^ Smartphone addiction is similar to internet addiction, and excessive use of smartphones can cause mental and behavioral problems 5,6 This study, aimed to investigate the quality of sleep in Pamukkale University students and to investigate its relationship with smartphone addiction.

## METHODS

This cross-sectional study was planned to be conducted with university students and at faculties in the campus in the 2017-2018 academic year. The dependent variable of the study was a poor sleep quality, and Pittsburgh Sleep Quality Index (PSQI) score higher than five indicates bad sleep quality. The minimum sample size was calculated as 1088 with a 95% confidence level and 80% power after examining the findings of other studies, with Open Epi (Open Source Epidemiologic Statistics for Public Health) Version 3.01.^7^,^8^ This number was increased by 20% to prevent possible problems that may occur. Hence, we aimed to reach 1306 students. A multistage cluster sampling method was used to recruit university students. Schools and faculties were accepted as clusters, and five faculties and one vocational school and students in all classes, which were selected randomly among seven faculties and two colleges were located in the Kinikli campus. There were a total of 14 faculties and three colleges in Pamukkale University Kinikli Campus. There were no students in some of the classes. Five faculties and one college with students from only normal education were selected among seven four-year faculties and two colleges with students from all grades. In the 2017-2018 academic year, the total number of students in seven four-year faculties and two colleges attending normal education was 20862.The questionnaire used in the study consisted of three sections that included a 30-item survey, a Smartphone Addiction Scale Short Form, and the Pittsburgh Sleep Quality Index. Smartphone Addiction Scale Short Form was developed by Kwon et al.^9^ Turkish validity and reliability was performed by Noyan et al. and Cronbach’s alpha coefficient was 0.867.^10^ The Likert type scale was scored from one to six. The lowest and highest scores to be obtained from the scale were 10 and 60, respectively. The risk of smartphone addiction increased in line with the scale score. The cut off score was identified as 31 for men and 33 for women in SAS-SF, which did not have a sub-scale. The Pittsburgh Sleep Quality Index which evaluated the sleep quality the last month. It was developed by Buysse et al.^7^ The Turkish scale validity and reliability study was conducted by Aaargun et al and Cronbach’s alpha coefficient was 0.80.^8^ A total score of above five which ranges between 0 and 21 indicates a poor sleep quality. Ethical committee approval was obtained from Pamukkale University Non-Interventional Clinical Research Ethics Committee numbered 60116787-020/8328(2018) and Statistical Package for Social Sciences (SPSS) for Windows 17.0 package program was used for analysis.

## RESULTS

Of the 1545 participant 56.8% were females and 43.2% were males. The mean age of students was 21.39 ± 2.21. Students’ total PSQI mean score was 6.17 ± 3.03, and the mean SAS-SF score was 28.63 ± 10.15 (Table-I). Of the students 52.4% had a PSQI total score higher than five and their sleep quality was poor. According to smartphone addiction cut-off points (31 points and above for men, 33 points and above for women), the frequency of addiction of students was 34.6% (35.9% for females and 33.0% for males). Six students who did not use smartphones were included in the group without smartphone addiction. In the study group, the frequency of having poor sleep quality was 64.5% for those with smartphone addiction and 45.9% for those without. The frequency of poor sleep quality was significantly higher in students with smartphone addiction compared to those without smartphone addiction (Table-II).

**Table-I:** Students’ PSQI and SAS-SF scores.

Variable	Number	Mean ±SD
PSQI total score	1545	6.17 ± 3.03
*SAS-SF score*		
Smartphone user	1539	28.63 ± 10.15
Not smartphone user	6	-

**Table-II:** Comparison of students’ sleep quality according to their smartphone addiction status.

*Variable*		*Sleep Quality*		*p*

		Poor Number (%)	Well Number (%)	
Smartphone Addiction	Yes	345 (64.5)	190 (35.5)	<0.001
	No *	464 (45.9)	546 (54.1)	

There was a positive correlation was found between the students PSQI total scores and SAS-SF scores (rho 0,242 p<0.001). A statistically significant linear relationship was found between students’ perceived financial status and their sleep quality (Table-III). Additionally, there were statistically significant relationships between students’ frequency of poor sleep quality and their smoking and alcohol use habits. Those who did not exercise had a higher rate of poor sleep quality than those who did at least 150 minutes of moderate-intensity physical activity or at least 75 minutes of vigorous-intensity physical activity per week or an equivalent combination of these, as recommended by WHO for the 18-64 age group (Table-IV). There was a significant linear relationship between the weekly consumption of coffee, caffeinated soft drinks, and energy drinks in the last month and sleep quality. Also there was a significant difference between the time of consuming caffeinated beverages and the students sleep quality (Table-III).

**Table-III:** Comparison of students’ sleep quality with other important variables.

Variable	*Sleep Quality*		p

	Poor Number (%)	Well Number (%)	

*Perceived income level*			
Income less than expenditures	195 (58.7)	137 (41.3)	0.016
Income is equal to expenditure	452 (51.1)	432(48.9)	
Income more than expenditures	156 (49.4)	160(50.6)	
*Smoking status*			
Never smoked	469 (48.7)	495 (51.3)	<0.001
Quit smoking	53(54.6)	44 (45.4)	
Occasionally smokes	104 (56.8)	79 (43.2)	
Regular smoker	183 (61.0)	117 (39.0)	
*Alcohol use in the past month*			
Never uses	584 (50.6)	570 (49.4)	0.002
Less than 1 day a week	137 (52.7)	123 (47.3)	
Consumes 1 day or more per week	85 (67.5)	41 (32.5)	
*Doing Regular Exercise*			
Yes	228 (47.2) 578	255 (52.8)	0.006
No	(54.7)	478 (45.3)	
*Physical activity for at least 150 minutes moderate or 75 minutes intense or an equivalent combination of the two in the last week*			
Yes	289 (48.1)	312 (51.9)	0.004
No	481(55.7)	382 (44.3)	
*Weekly coffee consumption*			
Not Consuming	91 (42.3)	124 (57.7)	<0.001
1-10 cups or cups / week	516 (52.1)	475 (47.9)	
> 10 glasses or cups / week>	176 (63.3)	102 (36.7)	
*Weekly caffeinated soft drink consumption*			
Not Consuming	205 (46.8)	233 (53.2)	0.002
1-5 cups or cans/week	420 (53.0)	372 (47.0)	
> 5 glasses or box/week>	143 (59.1)	99 (40.9)	
*Energy drink consumption in the last month*			
Never consumed in the last one month	684 (51.8)	636 (48.2)	0.047
Consumed 1-5 days in the last one month	96(56.8)	73 (43.2)	
Consumed more than 5 days in the past month	22 (66.7)	11 (33.3)	
*Hours of Caffeinated beverage consumption*			
During daytime	128 (43.2)	168 (56.8)	0.001
In the evening (after 18 o’clock)	210 (54.8)	173 (45.2)	
Both during the day and in the evening	467 (55.1)	381 (44.9)	
*Smartphone or tablet screen usage in the last One hour before bedtime*			
Yes	651 (53.9)	557 (46.1)	0.042
No	149 (47.5)	165 (52.5)	
Using smartphone in bed not using	66 (42.0)	91 (58.0)	<0.001
≤30 minutes/24 hours	247 (45.5)	296 (54.5)	
> 30 minutes/24 hours	443 (60.0)	295 (40.0)	
*Disease diagnosed by a physician*			
Yes	125 (59.0)	87 (41.0)	0.037
No	677 (51.2)	644 (48.8)	
*Regular medication use*			
Yes	93 (60.0)	62 (40.0)	.042
No	711 (51.4)	672 (48.6)	
*First-degree relative with sleep problems*			
Yes	181 (65.8)	94 (34.2)	<0.001
No	625 (49.4)	641 (50.6)	

	**Median**	**Median**	

Daily internet usage time(Hours/day)	4 (4.97 ± 2.96)	4 (4.61 ± 2.85)	.011
Daily smartphone usage time(hours/day)	5	4	<0.001

**Table-IV:** Binary logistic regression analysis results.

Variable (reference)	(B)	95% CI	p
*Smartphone addiction (No)*			
Yes	1.937	1.486-2.524	<0.001
*Perception of financial situation (Income is more than expenses)*			
Income is equal to expenditure	1.278	0.935-1.747	0.124
Income less than expenditures	1.769	1.212-2.581	0.003
*Smoking status (Never smoked)*			
Quit Smoking	1.173	0.695-1.981	0.551
Occasionally smokes	1.489	0.994-2.231	0.054
Regular smoker	1.510	1.064-2.142	0.021
*In the past one month, alcohol use (No)*			
Less than one day a week	1.087	0.769-1.536	0.638
Consumes one day or more per week	1.798	1.095-2.951	0.020
*Physical activity for at least 150 minutes of moderate or 75 minutes of intense or an equivalent combination of both in the last week (Yes)*			
No	1.696	1.319-2.180	<0.001
*Having first-degree relatives with sleep problems(No)*			
Yes	2.228	1.576-3.149	<0.001
*Using a smartphone in bed (No)*			
≤30 minutes/24 hours	1.062	0.687-1.644	0.786
> 30 minutes/24 hours	1.800	1.172-2.764	0.007

Daily internet usage differed according to students’ sleep quality: those with poor sleep quality had a significantly higher frequency of daily internet usage than others. The frequency of poor sleep quality was significantly higher in students using a smartphone or tablet within an hour before going to bed. The students with poor sleep quality had a significantly higher mean value of daily smartphone usage than others. The frequency of poor sleep quality increased in line with the frequency of smartphone usage, and there was a significant difference. The frequency of poor sleep quality significantly increased in the students who were diagnosed with a disease compared to those who were not in those who took regular medicines compared to those who did not, and in those who had a first-degree relative with sleep problems compared to those who did not (Table-IV).

In the multivariate analysis of factors related to the sleep quality of students, the variables which were significant in binary comparisons were evaluated using the binary logistic regression model. The risk of poor sleep quality was 1.937 times more significant in those with smartphone addiction than those without (Table-IV).

## DISCUSSION

Of the participants 52.4% had a poor sleep quality and one of the independent risk factors was smartphone addiction. Although smartphone addiction is a social problem, it should be considered in clinical practice. Studies conducted up to date with university students across the world have also reported a prevalence of poor sleep quality, which is consistent with our findings.^11^,^12^. This frequency was 59.4% in Lithuania^13^, 52.7% in Lebanon^14^, 55.8% in Ethiopia^15^, 33.8% in Taiwan^16^, 55% in the United States 17 and in Brazil 61.5% 18

A meta-analysis in Brazil found that children with low income levels had poorer sleep quality^19^ and these findings were in line findings of our study. Low perception of income level was an independent risk factor for poor sleep quality and this may be related to easy access to technological devices. Less poor sleep quality in students with high income perception may be related to the more conscious lifestyle created by financial opportunities in individuals.

Moreover, international scientific studies demonstrate the positive effects of exercise on sleep^20^,^21^ In this study, the students’ levels of engagement in moderate-intensity and vigorous-intensity physical activities over the last week were investigated and according to the binary logistic regression analysis, not doing physical activity at the level recommended by WHO was found to be an independent risk factor for poor sleep quality. This relationship was understandable considering the mental and physical positive effects of physical activity. In our study, the daily internet usage hours of students with poor sleep quality were significantly higher than others, and similarly, in studies conducted with university students in Taiwan^16^, the sleep quality of students with internet addiction was found to be significantly worse.

Daily smartphone usage hours of students with poor sleep quality were also significantly higher than others. So, the increase in daily smartphone usage time may lead to smartphone addiction and cause poor sleep quality. A study on students in Japan reported that playing computer games before going to sleep delays sleep^22^ We did not find a significant relationship between digital gaming and students sleep quality, which may be due to the use of smartphones for other purposes than digital gaming. We found that having first-degree relatives with sleep problems was a risk factor for poor sleep quality, which is also in line with the literature. In our study, the frequency of smartphone addiction was found to be 34.6%, and similarly to the findings of previous studies, we found that smartphone addiction to poses risks for poor sleep quality.

### Limitation of the study

Our strength was that we used valid and reliable scales for the study and investigated many variables related to sleep quality. Our limitation was that the study data were based on the participants statements.

## CONCLUSION

Smartphone addiction was found to be one of the risk factors for poor sleep quality. Students should be informed about sleep quality and smartphone addiction. During their university education and smartphone addiction should be questioned in clinical applications with poor sleep quality problems.

### Author Contribution:

**BO:** Did data collection, statistical analysis and manuscript writing.

**BO:** Is Responsible for the accuracy and integrity of the research.

**NMA:** Conceived, designed and editing of manuscript.
